# Supplementary material for: Full-spike deep mutational scanning helps predict the evolutionary success of SARS-CoV-2 clades
Source: bioRxiv. 2023 Nov 14:2023.11.13.566961. Preprint. [Version 1] doi: 10.1101/2023.11.13.566961 (PMC10680755; doi:10.1101/2023.11.13.566961)
Supplement: Supplement 1 [file NIHPP2023.11.13.566961V1-supplement-1.pdf]

bioRxiv preprint doi: https://doi.org/10.1101/2023.11.13.566961; this version posted November 14, 2023. The copyright holder for this preprint (which was not certified by peer review) is the author/funder, who has granted bioRxiv a license to display the preprint in perpetuity. It is made available under aCC-BY 4.0 International license.

Supplementary figures

bioRxiv preprint doi: https://doi.org/10.1101/2023.11.13.566961; this version posted November 14, 2023. The copyright holder for this preprint (which was not certified by peer review) is the author/funder, who has granted bioRxiv a license to display the preprint in perpetuity. It is made available under aCC-BY 4.0 International license.

a

Library	# of designed mutation	# of mutations in the final library	% of designed mutations in the final library	# of variants in the library
XBB.1.5 full spike Lib-1	6,865	9,236	98.8	74,561
XBB.1.5 full spike Lib-2	6,865	9,124	98.8	68,843
BA.2 full spike Lib-1	6,730	9,480	98.8	101,836
BA.2 full spike Lib-2	6,730	9,363	98.9	93,242
XBB.1.5 RBD Lib-1	3,888	3,970	99.2	70,365
XBB.1.5 RBD Lib-2	3,888	3,973	99.5	85,291

b

Parent clade	present in multiple descendant clades (# covered in libraries)	# of mutations present in only one descendant clade (# covered in libraries)
XBB	72 (72)	167 (163)
BA.5	57 (56)	110 (108)
BA.2	66 (65)	133 (121)

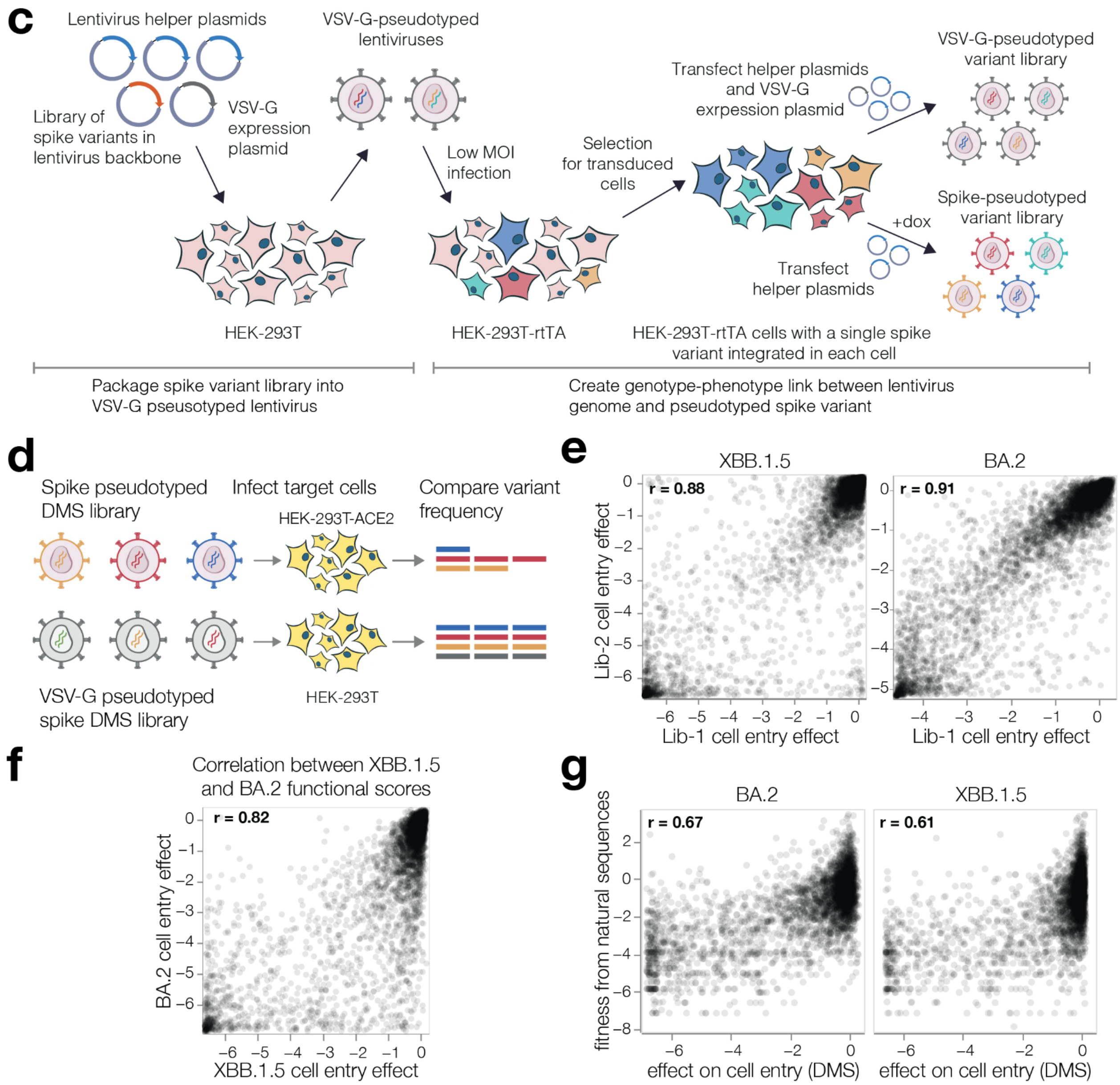

Extended Data Fig. 1: XBB.1.5 and BA.2 spike deep mutational scanning libraries

a, Number of targeted and final number of mutations and barcoded variants in the XBB.1.5 and BA.2 full spike and XBB.1.5 RBD pseudovirus-based deep mutational scanning libraries. **b,** Total number of unique mutations present in BA.2 and XBB descendant Pango clades and the number

bioRxiv preprint doi: https://doi.org/10.1101/2023.11.13.566961; this version posted November 14, 2023. The copyright holder for this preprint (which was not certified by peer review) is the author/funder, who has granted bioRxiv a license to display the preprint in perpetuity. It is made available under aCC-BY 4.0 International license.

of those mutations that are present in at least three barcoded variants in each replicate of the BA.2 or XBB.1.5 full spike libraries, which was the minimum number of occurrences we needed to make high-confidence estimates of the mutational effects on cell entry. **c,** Method for creating genotype-phenotype linked spike deep mutational scanning libraries, as previously described in Dadonaite et al. (2023)[4]. Lentivirus backbone plasmids encoding barcoded mutagenised spike genes together with helper and VSV-G expression plasmids are transfected into 293T cells to make VSV-G pseudotyped virus. These viruses are used to infect 293T-rtTA cells at MOI < 0.01 so that no more than one spike variant is integrated into each cell. Transduced cells are selected for lentiviral integration, and spike pseudotyped virus libraries are produced from these cells by transfecting helper plasmids in the presence of doxycycline to induce spike expression. In the absence of doxycycline and with added VSV-G expression plasmid, VSV-G pseudotyped virus libraries are also produced from the same cell lines; these VSV-G pseudotyped viruses are used to help estimate effects of spike mutations on cell entry as described in the next panel. **d,** Method used to measure effects of mutations in spike on cell entry. The ability of each spike variant to mediate cell entry is assessed by quantifying its relative frequency in 293T-ACE2 cells infected with spike-pseudotyped versus VSV-G pseudotyped libraries. **e,** Correlations between the effects of mutations on cell entry measured using each of the two independent full spike libraries of XBB.1.5 or BA.2. Throughout the rest of this paper, we report the mean value between the two libraries. **f,** Correlation between mutational effects on cell entry measured for the XBB.1.5 versus BA.2 full spike libraries. **g,** Correlation between mutational effects measured with the XBB.1.5 or BA.2 full spike libraries and fitness effects of those mutations estimated from actual human SARS-CoV-2 sequences[13].

bioRxiv preprint doi: https://doi.org/10.1101/2023.11.13.566961; this version posted November 14, 2023. The copyright holder for this preprint (which was not certified by peer review) is the author/funder, who has granted bioRxiv a license to display the preprint in perpetuity. It is made available under aCC-BY 4.0 International license.

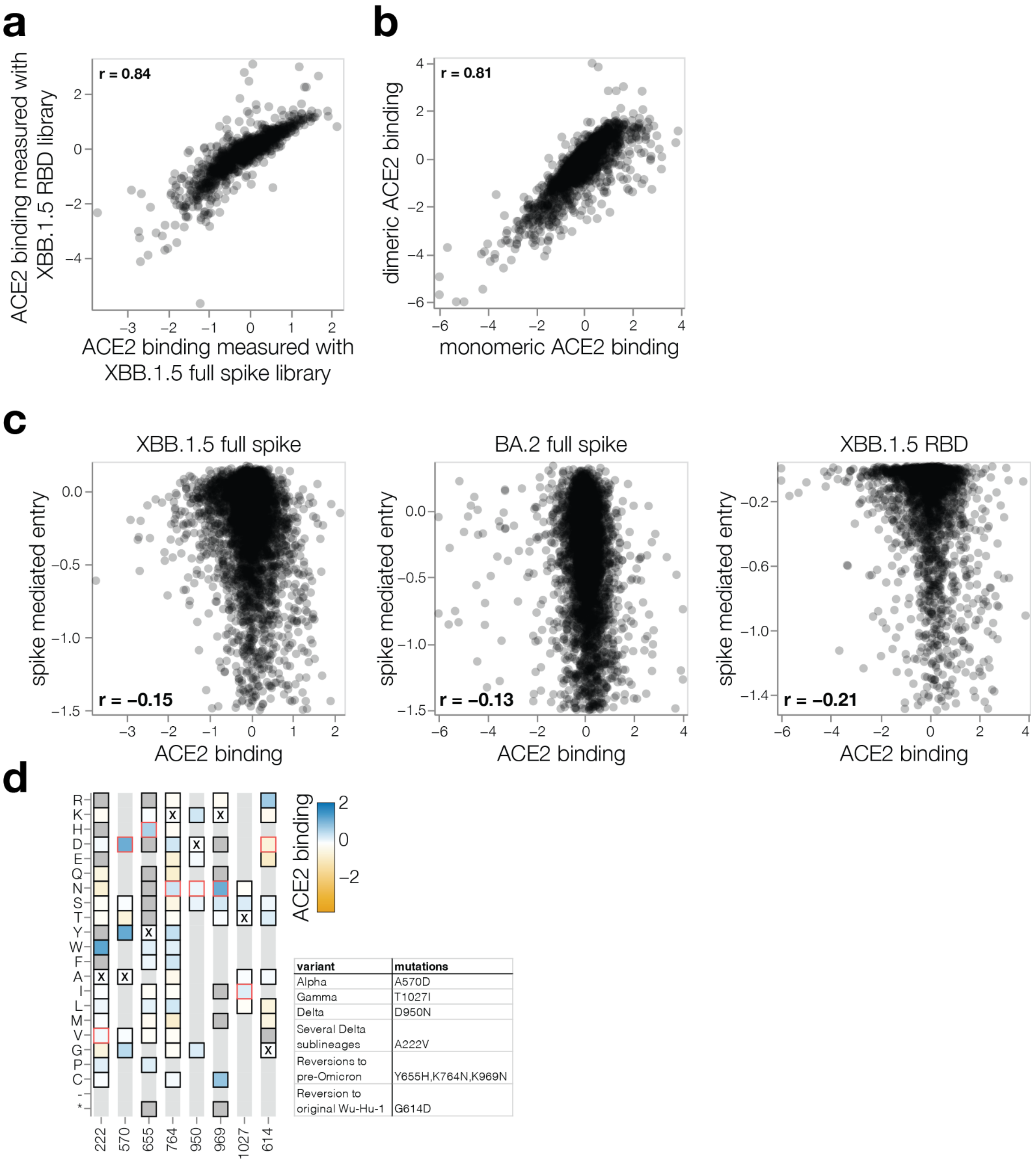

variant	mutations
Alpha	A570D
Gamma	T1027I
Delta	D950N
Several Delta sublineages	A222V
Reversions to pre-Omicron	Y655H,K764N,K969N
Reversion to original Wu-Hu-1	G614D

Extended Data Fig. 2: Correlations among measured mutational effects on ACE2 binding. **a,** Correlation between effects of mutations on ACE2 binding measured with XBB.1.5 full spike and XBB.1.5 RBD pseudovirus libraries. **b,** Correlation between effects of mutations on ACE2 binding measured using XBB.1.5 RBD pseudovirus library with monomeric and dimeric ACE2. Heatmaps

bioRxiv preprint doi: https://doi.org/10.1101/2023.11.13.566961; this version posted November 14, 2023. The copyright holder for this preprint (which was not certified by peer review) is the author/funder, who has granted bioRxiv a license to display the preprint in perpetuity. It is made available under aCC-BY 4.0 International license.

with the XBB.1.5 RBD pseudovirus measurements made using monomeric and dimeric ACE2 are at https://dms-vep.github.io/SARS-CoV-2_XBB.1.5_RBD_DMS/htmls/monomeric_ACE2_mut_effect.html and https://dms-vep.github.io/SARS-CoV-2_XBB.1.5_RBD_DMS/htmls/dimeric_ACE2_mut_effect.html, respectively **c,** Correlation between effects of mutations on ACE2 binding and spike-mediated cell entry for different libraries. **d,** ACE2 binding heat map showing key sites that have mutated in the past major SARS-CoV-2 variants. Specific variant mutations are highlighted in red outline. Table on the right indicates variants in which these mutations occurred. Interactive plot showing ACE2 binding for all mutations measured in spike is at https://dms-vep.github.io/SARS-CoV-2_XBB.1.5_spike_DMS/htmls/monomeric_ACE2_mut_effect.html.

bioRxiv preprint doi: https://doi.org/10.1101/2023.11.13.566961; this version posted November 14, 2023. The copyright holder for this preprint (which was not certified by peer review) is the author/funder, who has granted bioRxiv a license to display the preprint in perpetuity. It is made available under aCC-BY 4.0 International license.

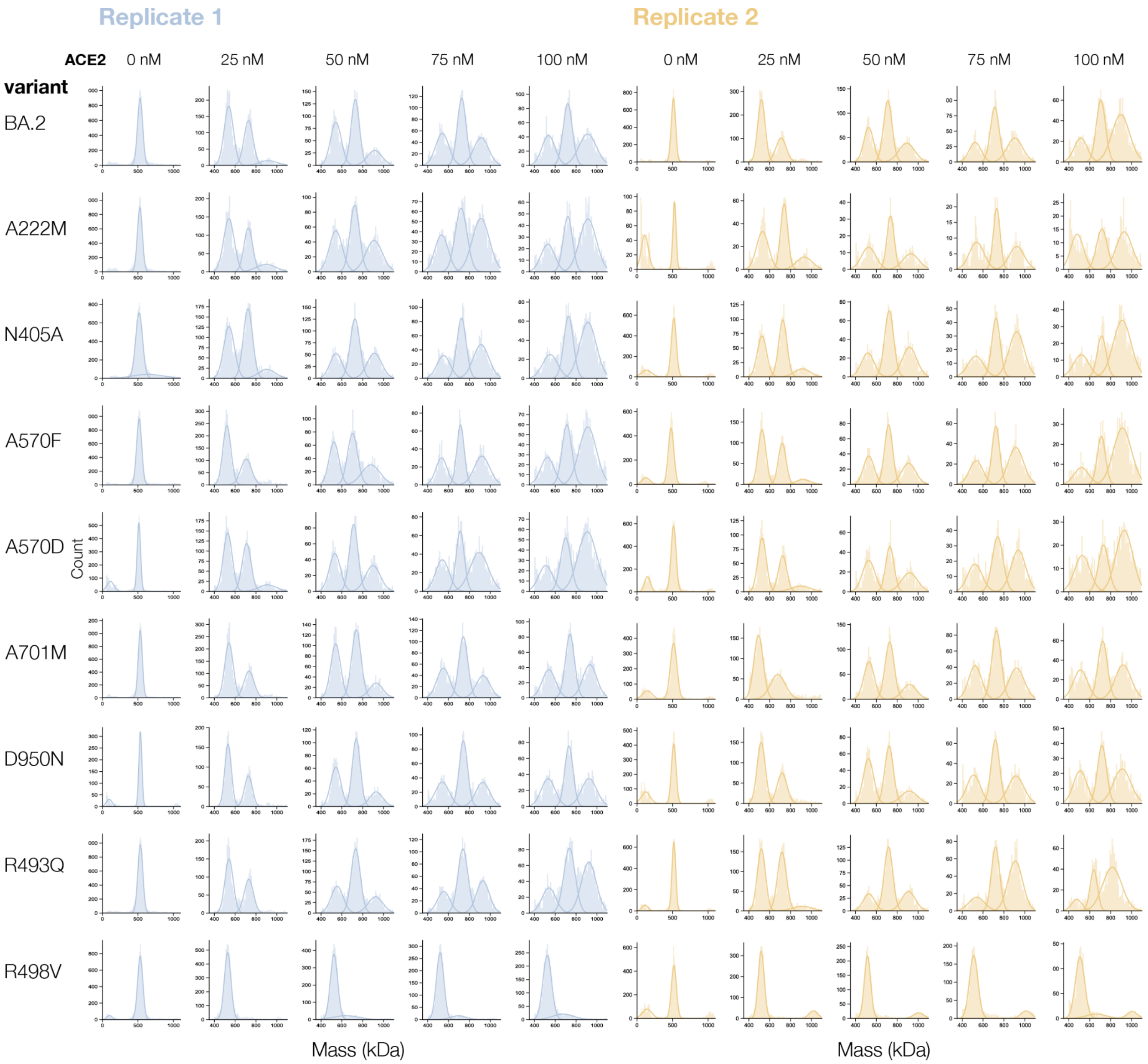

Extended Data Fig. 3: Mass photometry measurements for individual BA.2 spike variants
Spike molecular mass distributions measured using mass photometry for each biological replicate (blue and orange) corresponding to independent purification batches. Each row shows a BA.2 spike mutant and each column shows measurements at different ACE2 concentrations. In the absence of ACE2, some samples had a small peak to the left which may be a misfolded spike monomer[72] which was present only in some protein preparations and is excluded from Gaussian curve fitting in the presence of ACE2.

bioRxiv preprint doi: https://doi.org/10.1101/2023.11.13.566961; this version posted November 14, 2023. The copyright holder for this preprint (which was not certified by peer review) is the author/funder, who has granted bioRxiv a license to display the preprint in perpetuity. It is made available under aCC-BY 4.0 International license.

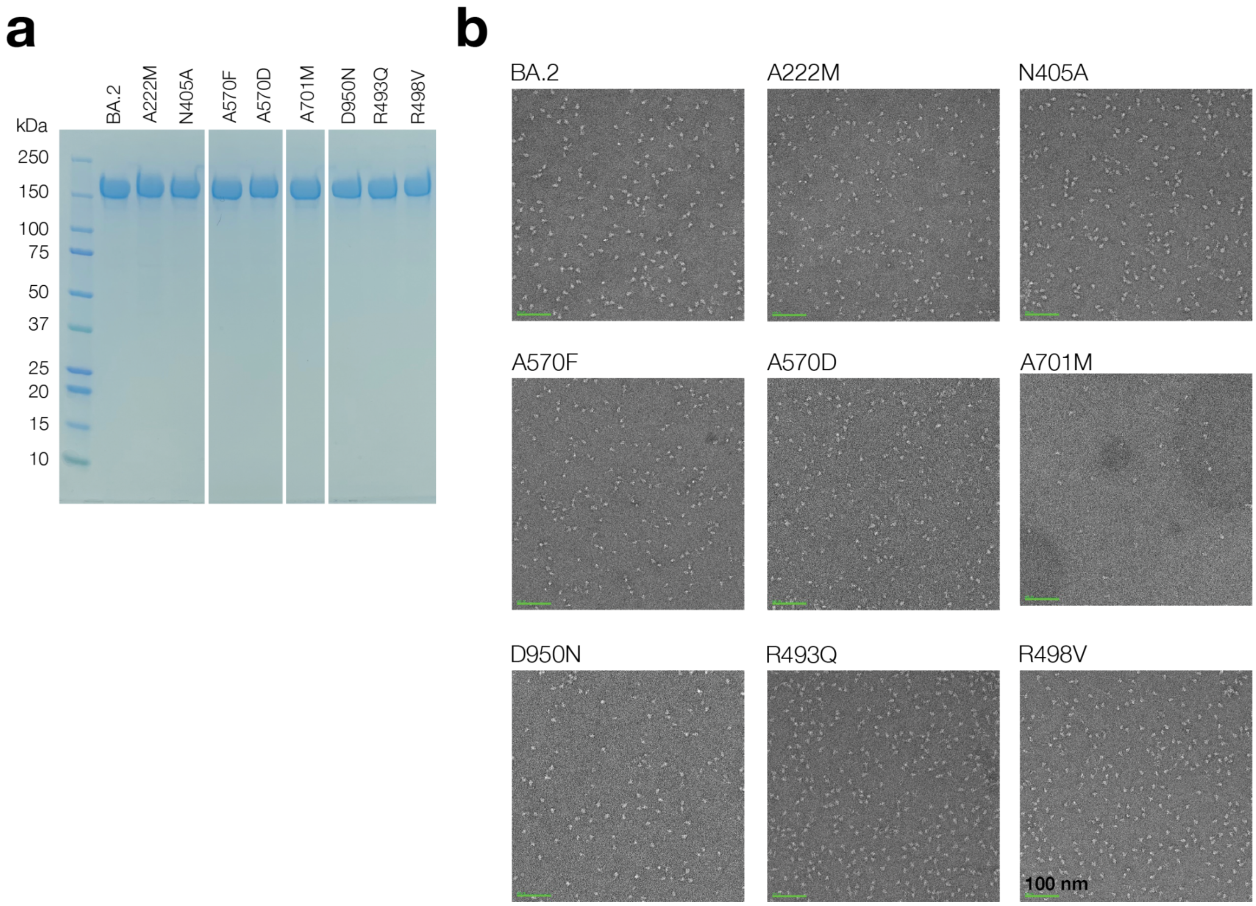

Extended Data Fig. 4: BA.2 and its mutant spike preparations

a, Reducing SDS-PAGE gel for purified BA.2 wildtype and mutant spike ectodomains. All constructs are pre-fusion stabilized with HexaPro mutations[28]. 3μg of purified protein loaded. Single major band for all samples confirms sample purity. **b,** Negative stain electron microscopy images for the purified spike mutants to confirm proper folding and monodispersity of the samples.

bioRxiv preprint doi: https://doi.org/10.1101/2023.11.13.566961; this version posted November 14, 2023. The copyright holder for this preprint (which was not certified by peer review) is the author/funder, who has granted bioRxiv a license to display the preprint in perpetuity. It is made available under aCC-BY 4.0 International license.

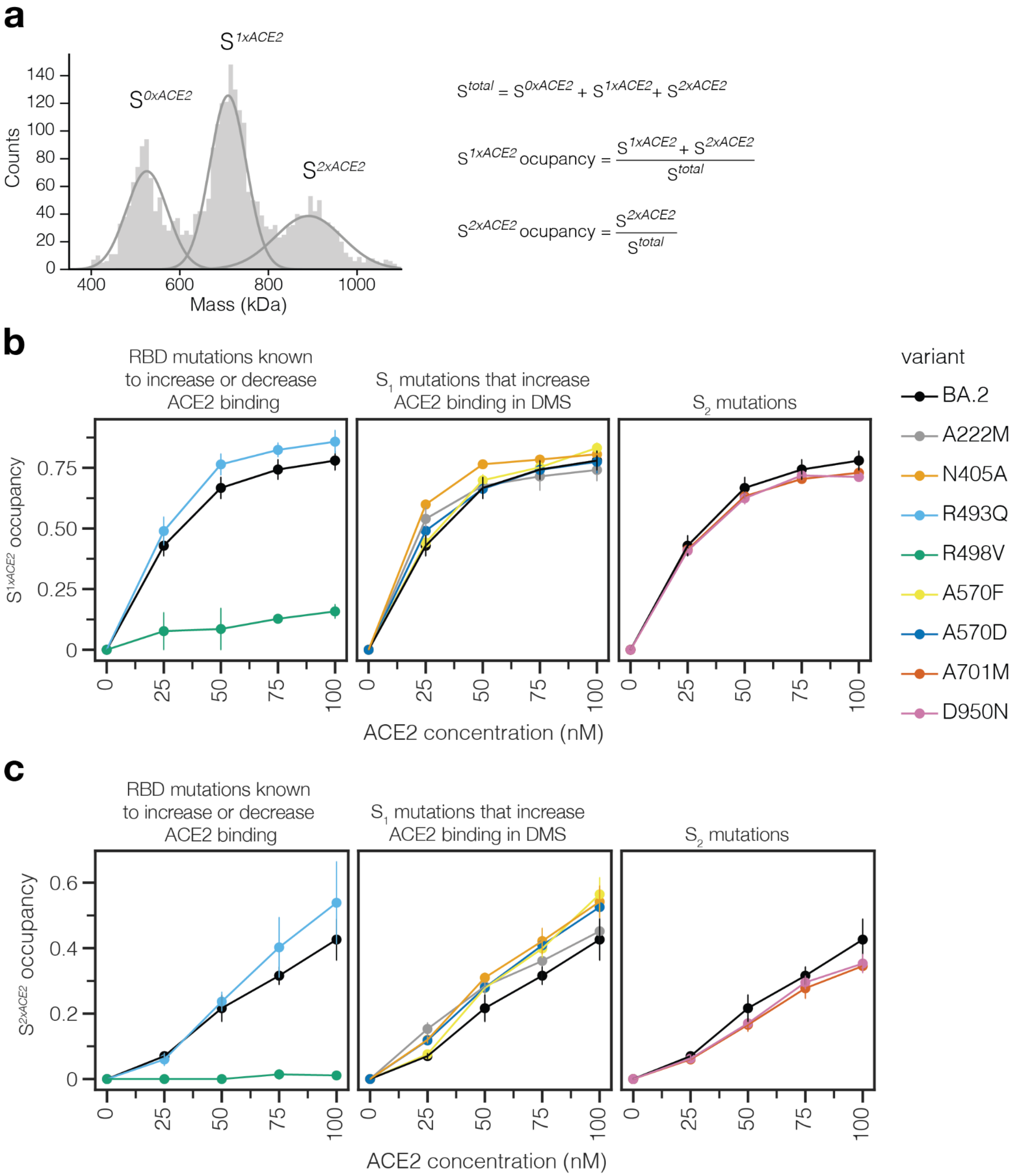

Extended Data Fig. 5: Mass photometry measurements for S_1 and S_2 occupancy
a, Illustration of Gaussian components for no (S^{0xACE2}) one (S^{1xACE2}) or two (S^{2xACE2}) ACE2-bound spike. S^{1xACE2} occupancy is the fraction of spikes bound by one ACE2 molecule and S^{2xACE2} is the fraction of spikes bound by two ACE2 molecules. **b,** S^{1xACE2} occupancy measured using mass photometry for different BA.2 spike mutants. **c,** S^{2xACE2} occupancy measured using mass photometry for different BA.2 spike mutants

bioRxiv preprint doi: https://doi.org/10.1101/2023.11.13.566961; this version posted November 14, 2023. The copyright holder for this preprint (which was not certified by peer review) is the author/funder, who has granted bioRxiv a license to display the preprint in perpetuity. It is made available under aCC-BY 4.0 International license.

bioRxiv preprint doi: https://doi.org/10.1101/2023.11.13.566961; this version posted November 14, 2023. The copyright holder for this preprint (which was not certified by peer review) is the author/funder, who has granted bioRxiv a license to display the preprint in perpetuity. It is made available under aCC-BY 4.0 International license.

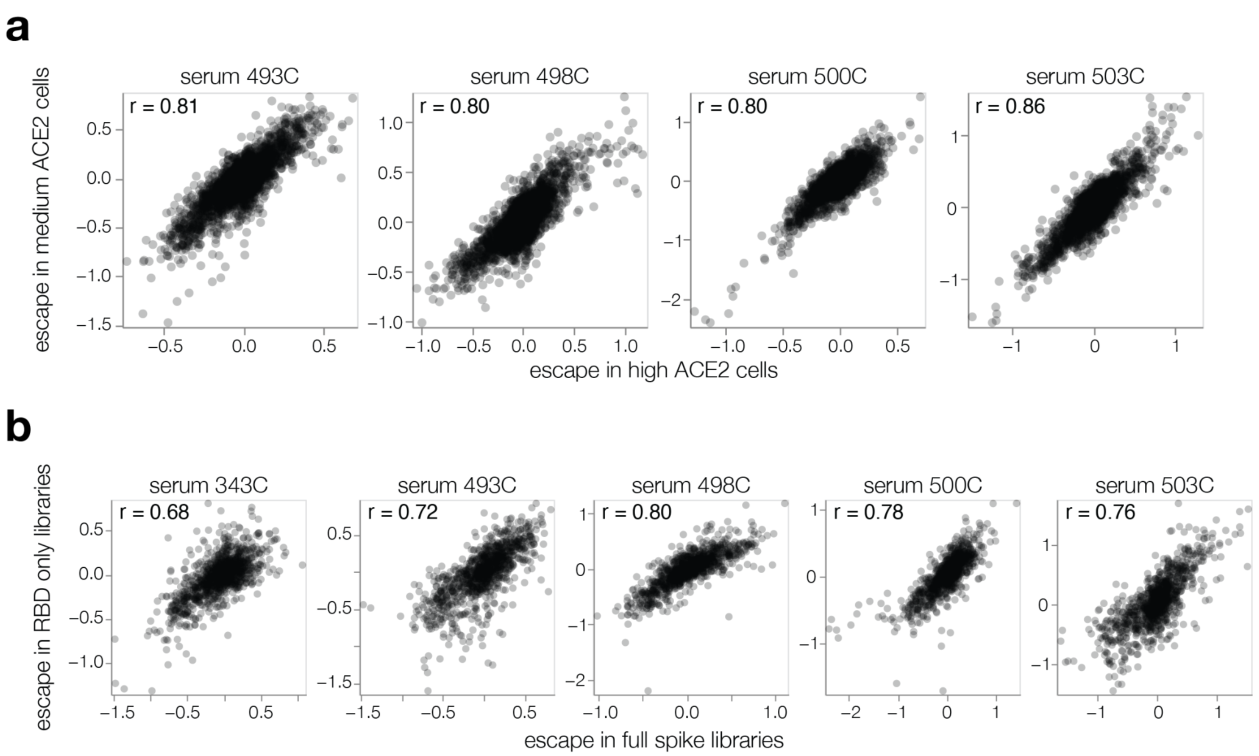

Extended Data Fig. 6: Correlation among serum escape mapping experiments

a, Correlation between mutation escape scores for experiments using the full-spike XBB.1.5 libraries performed on 293T cells expressing high or medium amounts of ACE2 for four sera. Note that the medium cells were used for all other figures shown in this paper. **b,** Correlation between mutation escape scores for mutations in the XBB.1.5 full spike and RBD-only libraries. See https://dms-vep.github.io/SARS-CoV-2_XBB.1.5_spike_DMS/htmls/compare_high_medium_ace2_escape.html and https://dms-vep.github.io/SARS-CoV-2_XBB.1.5_spike_DMS/htmls/compare_spike_rbd_escape.html for interactive versions of these scatter plots that also show line plots of per-site escape values for each serum.

bioRxiv preprint doi: https://doi.org/10.1101/2023.11.13.566961; this version posted November 14, 2023. The copyright holder for this preprint (which was not certified by peer review) is the author/funder, who has granted bioRxiv a license to display the preprint in perpetuity. It is made available under aCC-BY 4.0 International license.

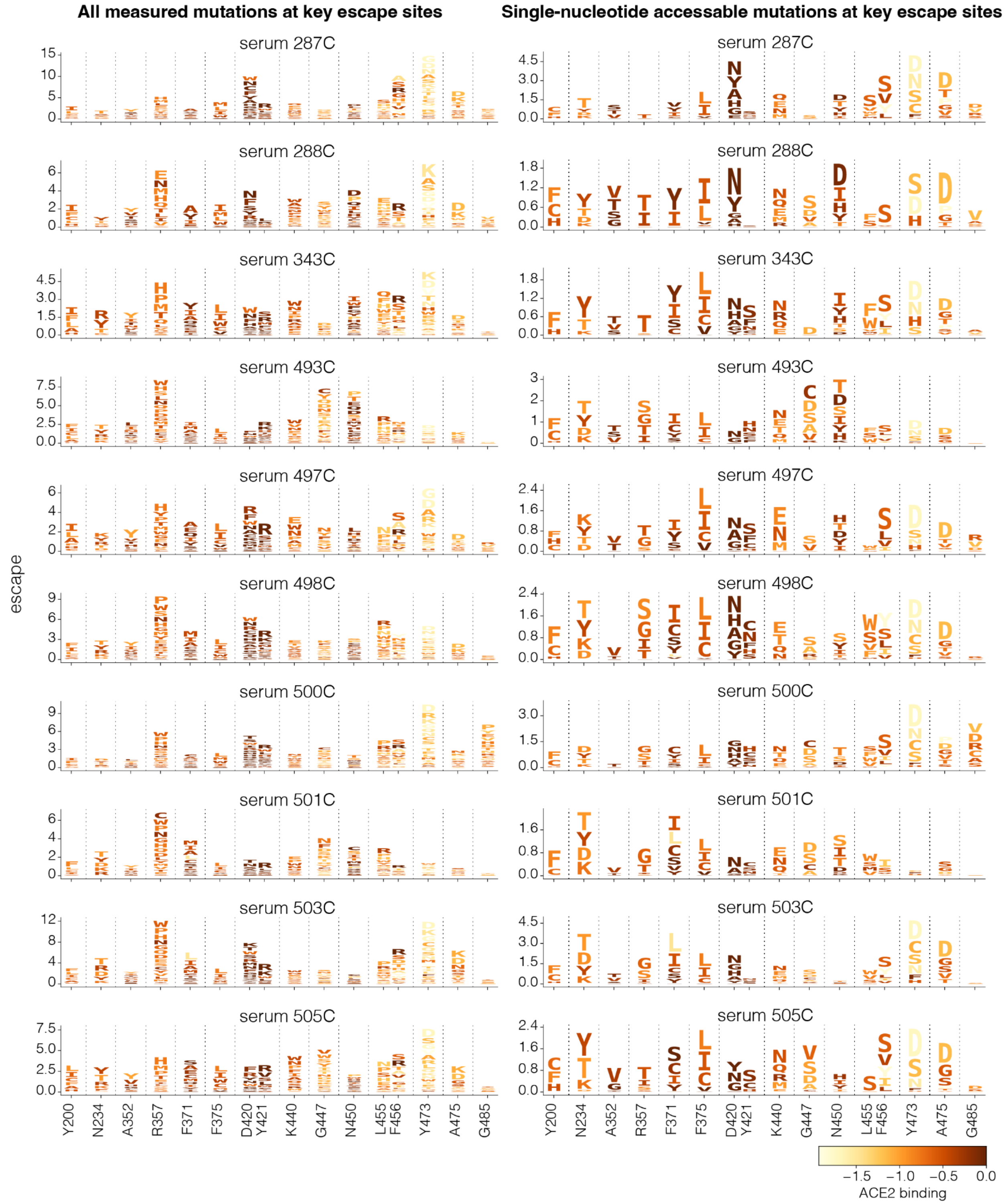

Extended Data Fig. 7: Escape at key sites for each serum

Logoplots showing XBB.1.5 spike escape at 16 highest escape sites for each of the 10 sera measured. Letter heights indicate the escape caused by mutation to that amino acid. Letters are colored light yellow to dark brown depending on mutation effect on ACE2 binding. Left: all mutations measured. Right: mutations accessible with a single-nucleotide substitution.

bioRxiv preprint doi: https://doi.org/10.1101/2023.11.13.566961; this version posted November 14, 2023. The copyright holder for this preprint (which was not certified by peer review) is the author/funder, who has granted bioRxiv a license to display the preprint in perpetuity. It is made available under aCC-BY 4.0 International license.

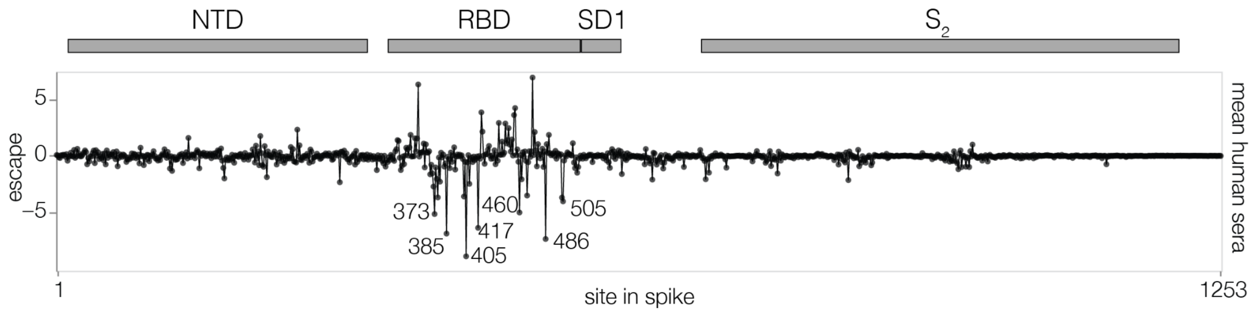

Extended Data Fig. 8: Mutations in XBB.1.5 spike that increase serum neutralization
Escape at each site in the XBB.1.5 spike averaged across the 10 sera from individuals with prior XBB* infections, showing negative as well as positive values (**Fig. 4** only shows positive values). Sites with negative escape in this plot are ones where many mutations make spike more sensitive to neutralization. Interactive plots with site and mutation-level escape are at https://dms-vep.github.io/SARS-CoV-2_XBB.1.5_spike_DMS/htmls/summary_overlaid.html (set 'floor escape at zero' at the bottom of the chart to false to show negative escape).

bioRxiv preprint doi: https://doi.org/10.1101/2023.11.13.566961; this version posted November 14, 2023. The copyright holder for this preprint (which was not certified by peer review) is the author/funder, who has granted bioRxiv a license to display the preprint in perpetuity. It is made available under aCC-BY 4.0 International license.

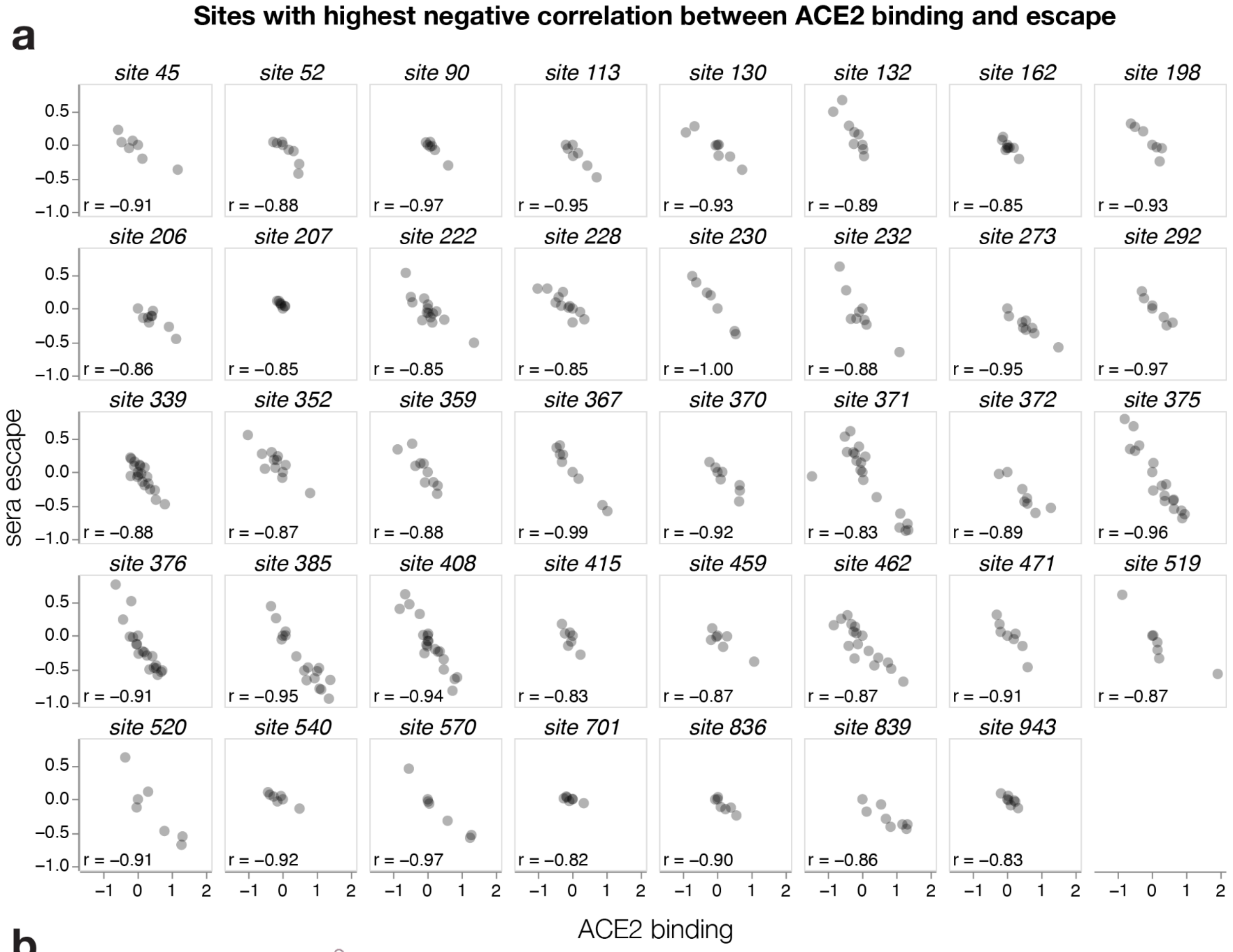

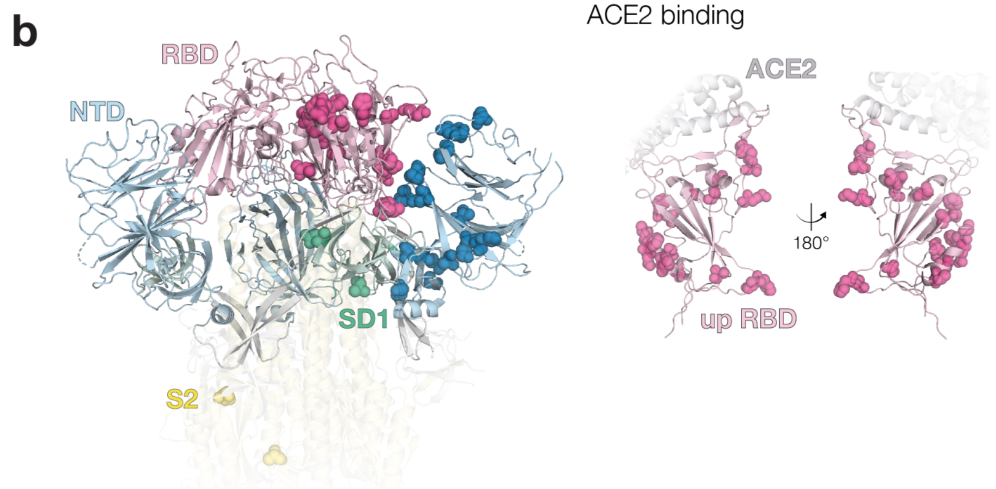

Extended Data Fig. 9: Sites with highest inverse correlation between ACE2 binding and serum escape

a, Correlation between ACE2 binding and serum escape for sites in XBB.1.5 spike. Only sites with at least 7 mutations measured and Pearson r < 0.82 are shown. **b,** Most sites with strongly negative correlations between mutational effects on ACE2 binding and escape are at positions that could plausibly impact the RBD conformation in the context of the full spike, since they tend to be

bioRxiv preprint doi: https://doi.org/10.1101/2023.11.13.566961; this version posted November 14, 2023. The copyright holder for this preprint (which was not certified by peer review) is the author/funder, who has granted bioRxiv a license to display the preprint in perpetuity. It is made available under aCC-BY 4.0 International license.

at the interface of the RBD and other spike domains. Left: all sites from **a** shown on spike structure as spheres. RBD is colored in light pink, NTD light blue, SD1 green and the S_2 subunit in yellow. Spheres are shown on only one chain for each domain for clarity (PDB ID: 8IOU). Right: RBD sites from **a** shown on RBD in up position engaged with ACE2. RBD is colored in light pink and ACE2 is gray.

bioRxiv preprint doi: https://doi.org/10.1101/2023.11.13.566961; this version posted November 14, 2023. The copyright holder for this preprint (which was not certified by peer review) is the author/funder, who has granted bioRxiv a license to display the preprint in perpetuity. It is made available under aCC-BY 4.0 International license.

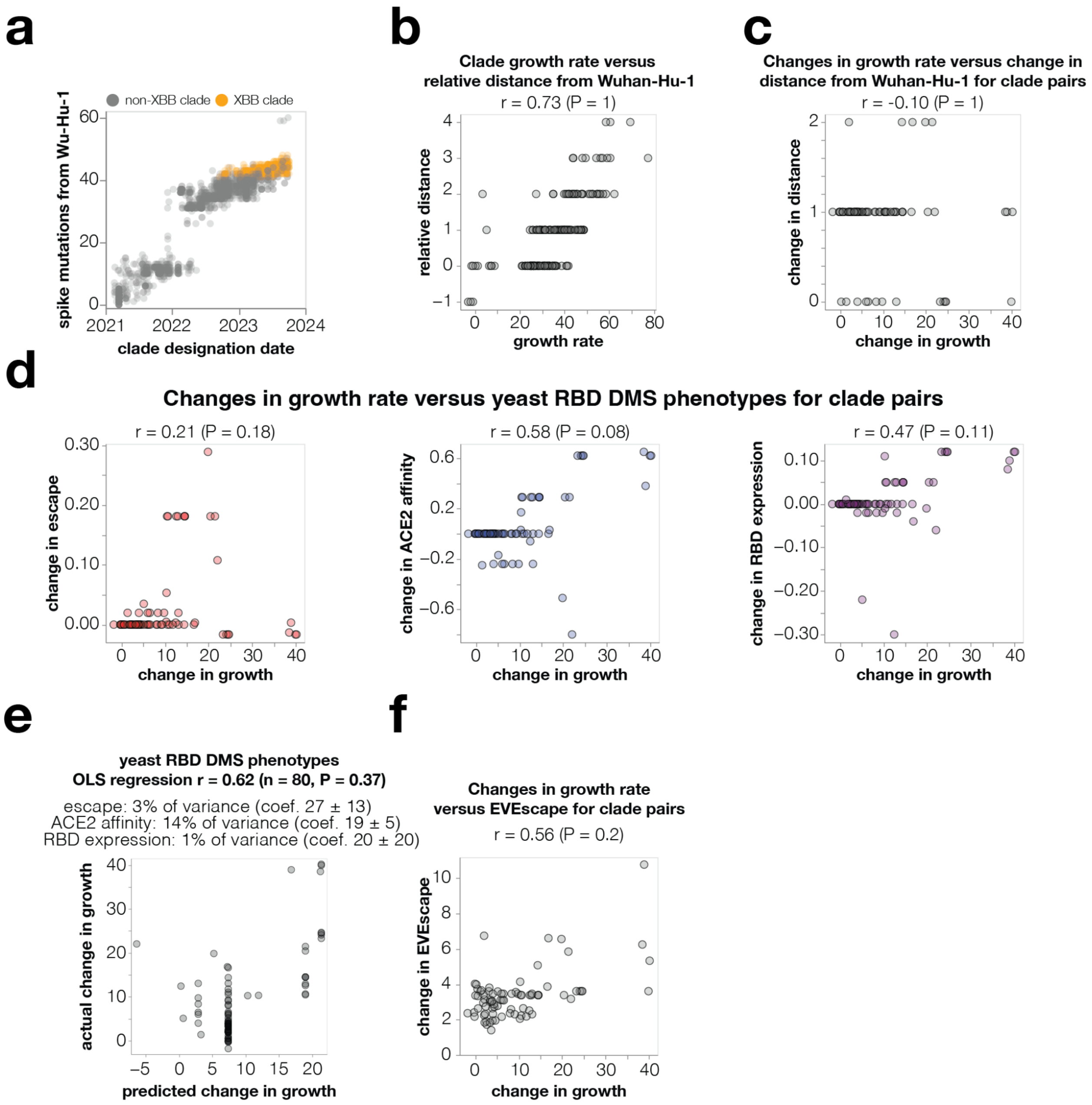

Extended Data Fig. 10: Correlations of clade growth and changes in clade growth with various other properties of spike

a, Number of spike amino-acid mutations relative to the early Wuhan-Hu-1 virus in all SARS-CoV-2 Pango clades versus the clade designation dates. XBB-descended clades are in orange. **b,** Because newer clades tend to have both more mutations and better growth, clade growth rate is trivially correlated with a clade's relative distance (number of spike mutations) from Wuhan-Hu-1. However, this correlation is not informative as it is already known that new clades tend to have more mutations. **c,** If we instead correlate the change in growth rate between parent-descendant clade pairs (Fig. 6b) with the change in spike mutational distance to Wuhan-Hu-1 there is no correlation, since this approach removes the co-variation with total mutation count. Therefore,

bioRxiv preprint doi: https://doi.org/10.1101/2023.11.13.566961; this version posted November 14, 2023. The copyright holder for this preprint (which was not certified by peer review) is the author/funder, who has granted bioRxiv a license to display the preprint in perpetuity. It is made available under aCC-BY 4.0 International license.

simple mutation counting is not informative for predicting changes in clade growth. **d,** Correlations of changes in clade growth with changes in site-level antibody escape, ACE2 affinity, and RBD expression measured for RBD mutations in yeast-display deep mutational scanning. **e,** Ordinary least-squares regression of changes in the RBD deep mutational scanning phenotypes versus changes in clade growth. **f,** Correlation of changes in the EVEscape score with changes in clade growth. Panels b-f are labeled with the Pearson correlation (r) and a P-value determined by computing how many randomizations of the mutational data yield correlations as large as the actual one.

bioRxiv preprint doi: https://doi.org/10.1101/2023.11.13.566961; this version posted November 14, 2023. The copyright holder for this preprint (which was not certified by peer review) is the author/funder, who has granted bioRxiv a license to display the preprint in perpetuity. It is made available under aCC-BY 4.0 International license.

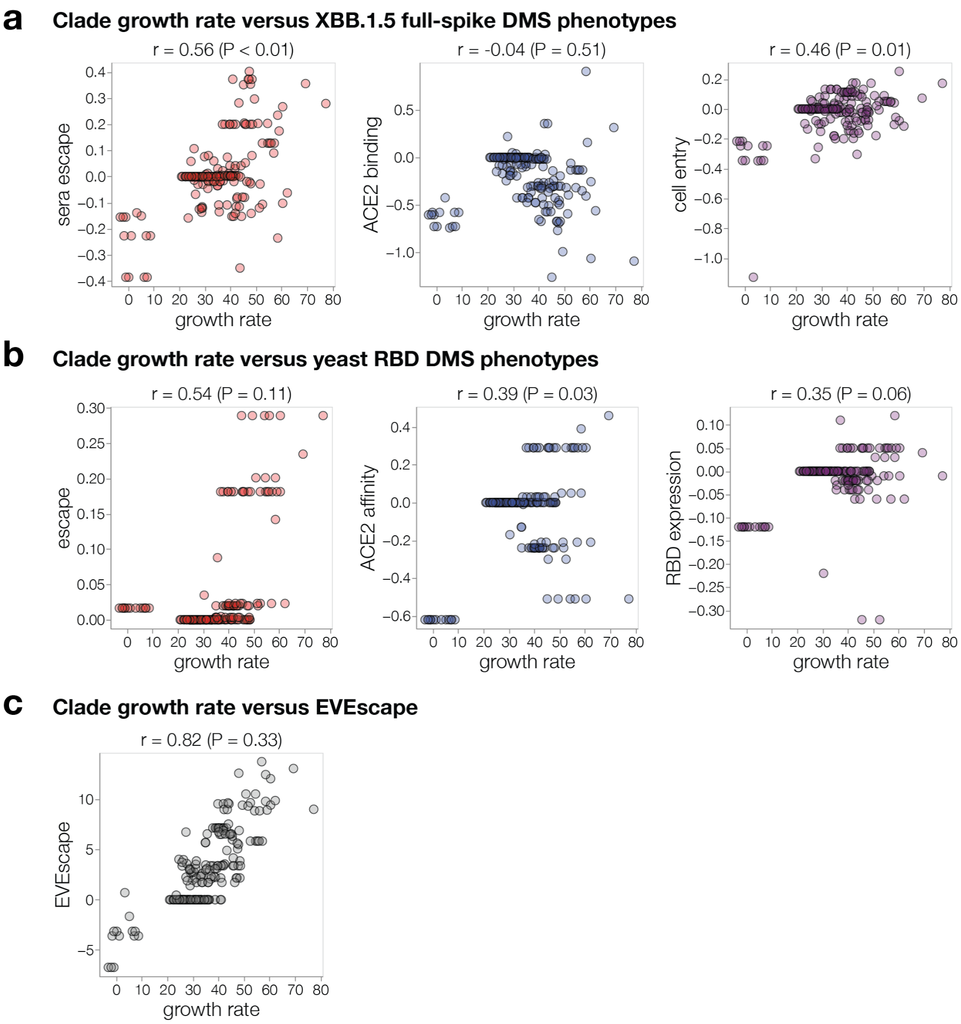

Extended Data Fig. 11: Correlations in absolute clade growth with absolute clade phenotypes

Correlations for **a,** the phenotypes measured by the full spike deep mutational scanning in the current paper; **b,** the phenotypes measured in yeast display RBD deep mutational scanning; **c,** predicted by the EVEscape method. These plots differ from **Fig. 6c** and **Extended Data Fig. 10d,f** in that they show the correlations in absolute clade growth with the absolute clade phenotypes, rather than comparing the changes in both for each parent-descendant clade pair. Absolute clade phenotypes are computed as the sum of mutation effects. The P-values above the plots are the fraction of times the correlation is greater than that for the actual data after randomizing the phenotypic effects among mutations. Note that the correlations are no longer at all reflective of the P-values for the reasons noted in the main text and **Extended Data Fig. 10c**—phylogenetic correlations, and the fact that new clades have both more mutations and higher growth so that any "phenotype" that amounts to counting mutations gives a correlation in these plots. For this reason, comparing changes in clade growth to changes in spike phenotypes as done in **Fig. 6c** and **Extended Data Fig. 10d,f** is the correct approach to test whether a method can actually predict which new clades will be successful.

bioRxiv preprint doi: https://doi.org/10.1101/2023.11.13.566961; this version posted November 14, 2023. The copyright holder for this preprint (which was not certified by peer review) is the author/funder, who has granted bioRxiv a license to display the preprint in perpetuity. It is made available under aCC-BY 4.0 International license.

bioRxiv preprint doi: https://doi.org/10.1101/2023.11.13.566961; this version posted November 14, 2023. The copyright holder for this preprint (which was not certified by peer review) is the author/funder, who has granted bioRxiv a license to display the preprint in perpetuity. It is made available under aCC-BY 4.0 International license.

Serum sample	Sex	Race	Age	Number of infections	Number of vaccine and booster doses	1st infection symptom date	2nd infection symptom date	3rd infection symptom date
287C	Female	White	57	3	5	Sep-2021	May-2022	Mar-2023
288C	Male	White	56	3	5	Aug-2021	May-2022	Mar-2023
343C	Female	Asian	19	2	4	Jan-2022	Apr-2023	
493C*	Male	White	28	1	2	Jan-2023		
497C	Female	White	48	2	4	Apr-2022	Mar-2023	
498C*	NA	NA	NA	1	5	Dec-2022		
500C	Male	Asian	63	1	5	Mar-2023		
501C	Male	White	53	1	5	Mar-2023		
503C	Male	White	51	1	6	May-2023		
505C*	Female	Middle Eastern	41	1	3	Feb-2023		

* sequencing confirmed infection. 493C and 505C had XBB.1.5 infection and 498C had XBB infection.

Supplementary Table 1: Information on sera used in this study

Sera selected for this study was from individuals who either had a confirmed XBB* infection (marked by * in the table above) or had the last recorded infection during the period when XBB or its descendant lineages were the most common circulating variants in Washington state. In February 2023 70% of sequenced cased were confirmed XBB or its descendant lineages and between March and May this number grew from 88% to 97% according to the samples sequenced at University of Washington Virology labs[73]. NA indicates information not available for that individual.
